# iRGD‐Targeted Physalis Mottle Virus Like Nanoparticles for Targeted Cancer Delivery

**DOI:** 10.1002/smsc.202300067

**Published:** 2023-06-27

**Authors:** Krister J. Barkovich, Zhongchao Zhao, Nicole F. Steinmetz

**Affiliations:** ^1^ Department of Radiology University of California, San Diego San Diego CA 92093 USA; ^2^ Department of NanoEngineering University of California, San Diego San Diego CA 92093 USA; ^3^ Center for Nano-ImmunoEngineering University of California, San Diego San Diego CA 92093 USA; ^4^ Department of Bioengineering University of California, San Diego San Diego CA 92093 USA; ^5^ Institute for Materials Discovery and Design University of California, San Diego San Diego CA 92093 USA; ^6^ Moores Cancer Center University of California, San Diego San Diego CA 92093 USA; ^7^ Center for Engineering in Cancer, Institute for Engineering in Medicine University of California, San Diego San Diego CA 92093 USA

**Keywords:** cancer, doxorubicin, drug delivery, iRGD, nanomedicine, plant viruses

## Abstract

Nanomedicine provides a promising platform for the molecular treatment of disease. An ongoing challenge in nanomedicine is the targeted delivery of intravenously administered nanoparticles to particular tissues, which is of special interest in cancer. Herein, the conjugation of iRGD peptides is shown, which specifically targets tumor neovasculature, to the surface of Physalis mottle virus (PhMV)‐like nanoparticles, leading to rapid cellular uptake in vitro and tumor homing in vivo. It is then shown that iRGD‐targeted PhMV loaded with the chemotherapeutic doxorubicin shows increased potency in a murine flank xenograft model of cancer. The results validate that PhMV‐like nanoparticles can be targeted to tumors through iRGD‐peptide conjugation and suggest that iRGD‐PhMV provides a promising platform for the targeted delivery of molecular cargo to tumors.

## Graphical abstract

Nanomedicine provides a promising platform for the molecular treatment of disease; however, the tissue‐specific targeting of intravenously delivered nanoparticles remains a challenge. The conjugation of iRGD peptides, which target tumor neovascular, to the surface of Physalis mottle virus like nanoparticles improves nanoparticle delivery to tumors for both cancer imaging and drug delivery purposes.

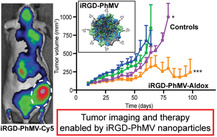

## 1 Introduction

Cancer is the second leading cause of death in the United States, causing greater than 600 000 deaths in 2020.^[^ ^1^ ^]^ Our growing understanding of the molecular mechanisms of cancer has led to an ever‐expanding arsenal of molecular targeted therapy. However, the clinical translation of many promising therapies has been hampered by poor efficacy, toxicity, and off‐target effects, perhaps due to the imprecise delivery of these therapeutics to the tissue or organ of interest.^[^ ^2^, ^3^ ^]^ As generalizable strategies for the targeted delivery of cargo to specific tissues remains a challenge, there is increasing research into the use of nanomaterials as carriers for the targeted delivery of chemotherapeutics, synthetic nucleic acids, and imaging reagents to tumors.^[^ ^4^, ^5^ ^]^ While data demonstrate tumor homing of nanoparticles (NPs) through various targeting approaches, a meta‐analysis of the data indicates that only 0.7% of intravascularly administered NPs are delivered to solid tumors.^[^ ^6^ ^]^ Hurdles to tissue‐specific delivery of NPs are numerous and include nonspecific uptake by the mononuclear phagocyte system (MPS) and endothelial and cellular barriers at the tumor.^[^ ^7^ ^]^

Multiple different nanocarrier platforms are currently under development and include lipid‐based, polymeric, and inorganic NPs, as well as naturally occurring and engineered viruses or viral vectors,^[^ ^8^ ^]^ each of which has its own advantages and disadvantages in terms of carrying capacity, biodistribution, and facility of manipulation.^[^ ^9^ ^]^ In comparison with synthetic NPs, viral NPs are protein‐based nanostructures with high biocompatibility (i.e., when using noninfectious viruses), structural uniformity, and ease of synthesis and manipulation through cell culture, fermentation, or molecular farming.^[^ ^10^ ^]^ Virus‐like particles (VLPs), proteinaceous NPs derived from the coat protein of viral capsids, lack the genomic nucleic acids of other viral NPs and are therefore noninfectious.^[^ ^11^ ^]^ While the immunogenicity and safety concerns for mammalian virus‐based NPs have limited the translation of these technologies into the clinic, plant virus NPs, and VLPs are an emerging alternative.^[^ ^12^ ^]^ Virus‐based NPs can be easily functionalized through genetic manipulation and reactive amino acids to carry chemotherapeutics, synthetic genes, and imaging reagents,^[^ ^13^, ^14^, ^15^ ^]^ and functionalized to tune pharmacokinetics.^[^ ^16^ ^]^ Therefore in this work, we turned toward the study of a plant VLP, engineered to home to tumors. While the aforementioned meta‐analysis was very comprehensive, it lacked the analysis of data for VLPs or viral vectors. To fill this knowledge gap, we chose a plant VLP combined with a universal targeting strategy. Specifically, we used VLPs derived from the plant virus Physalis mottle virus (PhMV), a +ssRNA virus from the family *Tymoviridae* that forms a ≈30 nm‐sized icosahedral capsid from 180 identical coat proteins, and can be expressed and purified from *Escherichia coli* as a monodisperse and stable VLP.^[^ ^17^ ^]^ PhMV VLPs can be functionalized internally with chemotherapeutics and imaging reagents^[^ ^18^ ^]^ and externally with targeting peptides.^[^ ^19^ ^]^

One of the six hallmarks of cancer is sustained angiogenesis,^[^ ^20^ ^]^ the process by which new capillaries sprout and branch from existing vasculature.^[^ ^21^ ^]^ In adults, there are few tissues with physiologic angiogenesis, so this molecular signature is relatively specific for malignancy and injured tissues.^[^ ^22^ ^]^ As such, tumors can be targeted with relative specificity by directing NPs toward new vasculature, and several biologics targeting this process have been FDA‐approved for the treatment of metastatic GI malignancies and renal cell carcinoma.^[^ ^23^ ^]^ Peptides with an arginine–glycine–aspartate (RGD)‐motif have been shown to display high affinity for αvβ3 and αvβ5 integrins, which are upregulated on angiogenic endothelial cells.^[^ ^24^ ^]^ The iRGD peptide, which includes a C‐end rule (CendR) motif within a cyclic RGD peptide, undergoes a proteolytic cleavage to reveal a neuropilin‐1 (NRP‐1) binding fragment that stimulates uptake within the tumor stroma.^[^ ^25^ ^]^ Cyclic RGD and iRGD peptides have been used for delivery of a wide range of cargo to tumor models, including therapeutics, nanoparticles, and imaging reagents.^[^ ^26^, ^27^, ^28^ ^]^ Data from other nanoparticle systems indicate that conjugation to iRGD peptides leads to a two‐ to eightfold increase in tumor localization of oncolytic adenovirus NPs and aggregated albumin‐based NPs, respectively,^[^ ^25^, ^29^ ^]^ although increased intratumoral localization of lipid bilayer‐coated silica NPs was not seen with iRGD conjugation.^[^ ^28^ ^]^ Here, we assess if iRGD peptides can be used for targeted delivery of PhMV VLPs to tumors. Specifically, we analyze whether iRGD‐PhMV VLPs are taken up by cells in vitro and show increased intratumoral localization in vivo. We then evaluate if iRGD‐PhMV VLPs loaded with the chemotherapeutic doxorubicin can be used for targeted cancer treatment.

## 2 Results

### 2.1 Preparation and Characterization of iRGD‐Conjugated PhMV Nanoparticles

**Figure 1:**
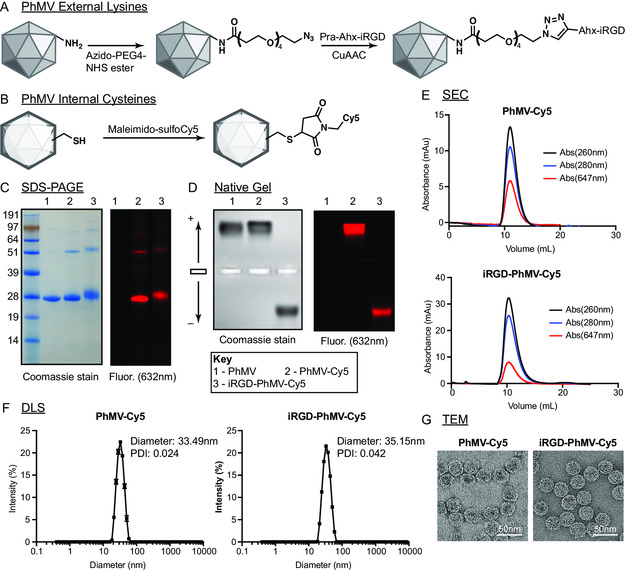
Synthesis of PhMV‐iRGD conjugates. A) Scheme for conjugation of iRGD to the external lysines of PhMV using NHS‐ester chemistry and copper (I)‐catalyzed azide–alkyne cycloaddition (CuAAC). B) Scheme for conjugation of sulfoCy5 to internal cysteines of PhMV using maleimide chemistry. C–G) Characterization of iRGD‐PhMV‐Cy5 and PhMV‐Cy5 using SDS‐PAGE (C), native gel electrophoresis (D), size‐exclusion chromatography (E), dynamic light scattering (F), and transmission electron microscopy (G).

PhMV‐like nanoparticles were prepared by expressing the PhMV coat protein in *E. coli*, as previously described,^[^ ^18^ ^]^ and were found to be monodisperse and homogenous (Figure S1, Supporting Information). To conjugate iRGD peptides to the external surface of PhMV, we functionalized the external lysines with a bifunctional NHS‐ester‐azide linker that was coupled to iRGD functionalized with an N‐terminal propargylglycine through copper (I)‐catalyzed azide–alkyne cycloaddition^[^ ^30^, ^31^ ^]^ (**Figure** **1**A). The internal compartment of PhMV was loaded with the near‐IR fluorescence (NIRF) dye sulfo‐Cy5 using maleimide–cysteine chemistry to allow for NP tracking by fluorescence imaging (Figure 1B). Conditions were selected to load a small quantity of dye per coat protein to avoid internal fluorescence quenching,^[^ ^18^ ^]^ and denatured coat proteins and whole particles showed robust NIRF fluorescence (Figure 1C,D). PhMV VLPs offer 180 internal cysteines and 720 external lysines for bioconjugation; based on absorbance measurements approximately 10 sulfo‐Cy5 dyes were conjugated per VLP; at this density quenching but providing sufficient signal for imaging. Native gel electrophoresis, SDS‐PAGE, and size‐exclusion chromatography (SEC) confirmed covalent attachment of the dye as Cy5 co‐migrated with VLPs or denatured coat proteins (Figure 1C–E).

The final product, iRGD‐PhMV‐Cy5 (and an iRGD‐free control, PhMV‐Cy5), was structurally sound and overall matched the NP characteristics of the unmodified VLPs. Monodisperse and homogeneous NP preparations of iRGD‐PhMV‐Cy5 were detected by native gel electrophoresis, SEC, and dynamic light scattering (DLS) (Figure 1D–F). SEC showed the characteristic elution profile (≈11 mL from Superose 6 increase 10/300 GL column) with Cy5 and VLP coeluting; aggregation, broken VLPs, or free CP was not detected. Transmission electron microscopy (TEM) was used to confirm the structural integrity of the NPs (Figure 1G). DLS and TEM were in agreement showing NPs measuring ≈30 nm in diameter. The marked mobility change between PhMV VLPs and iRGD‐PhMV‐Cy5 by native gel electrophoresis (Figure 1D) is explained by the neutralization of surface lysines through reaction with the NHS‐ester‐azide linker.

The amount of iRGD peptide loaded onto PhMV could be altered by adjusting the molar ratio of peptide per PhMV coat protein (Figure S2, Supporting Information), with a near linear relationship observed at low concentration (Figure S2B,C, Supporting Information). Therefore, we also generated a set of nanoparticles with a range of concentration of surface‐bound iRGD peptides: in addition to iRGD‐PhMV‐Cy5, which was synthesized using an equimolar ratio of iRGD peptide to PhMV coat proteins, we also generated particles with 0.5 and 2.0 iRGD peptides per coat protein, referred to as iRGD‐PhMV‐Cy5‐0.5 and iRGD‐PhMV‐Cy5‐2.0, respectively (Figure S3, Supporting Information).

### 2.2 αv Integrin‐Binding Properties of iRGD‐PhMV Nanoparticles

**Figure 2:**
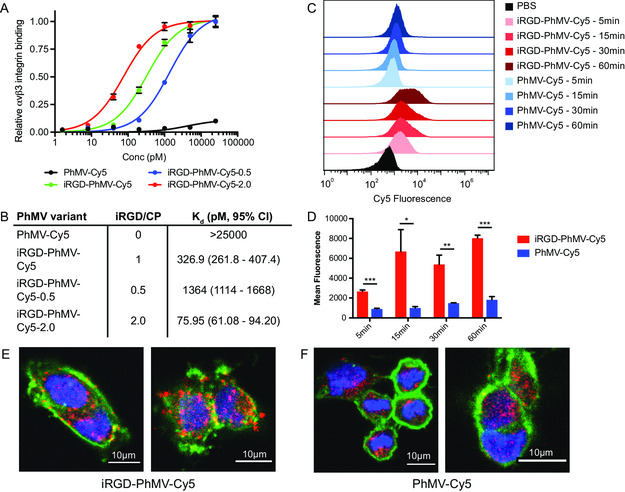
iRGD‐PhMV binds tightly to αvβ3 integrins and is rapidly taken up by A2780 cells. A,B) Binding curves (A) and calculated *K*_d_ (B) of iRGD‐PhMV‐Cy5 panel in αvβ3 integrin‐binding ELISA. C,D) iRGD‐PhMV‐Cy5 uptake by A2780 cells after 5, 15, 30, and 60 min, as measured by flow cytometry (C) with quantification (D). E,F) Confocal imaging of A2780 cells after 10 min incubation with iRGD‐PhMV‐Cy5 (E) or PhMV‐Cy5 (F). The error bars represent the standard error of the mean (S.E.M.) of three replicates. Statistical analysis by two‐tailed *t*‐test (*: *p* < 0.05, **: *p* < 0.01, ***: *p* < 0.001).

An enzyme‐linked immunosorbent assay (ELISA) was next performed to assess whether the iRGD peptides on the surface of PhMV VLPs were appropriately positioned and maintained affinity for αv integrins (**Figure** **2**A). iRGD‐PhMV‐Cy5 NPs bound αvβ3 integrins with picomolar affinity (Figure 2B). This affinity was dose‐dependent with the surface concentration of iRGD peptide, and iRGD‐PhMV‐Cy5‐2.0 displayed a sub‐100 pm affinity for αvβ3 integrin, while non‐iRGD functionalized NPs show minimal binding.

### 2.3 Cancer Cell Binding Properties of iRGD‐PhMV Nanoparticles

PhMV VLPs have previously been shown to be internalized by cultured cells through endocytosis into the endolysosomal compartment.^[^ ^18^ ^]^ To assess whether iRGD peptide conjugation to the VLPs alters the rate or fate of cellular uptake of the VLPs, A2780 cells were incubated with iRGD‐PhMV‐Cy5 or PhMV‐Cy5 and analyzed using flow cytometry. We observed a fivefold increase in the level of uptake of iRGD‐conjugated PhMV as compared to control at all timepoints under 1 h (Figure 2C,D). A twofold increase or decrease in RGD‐peptide concentration on the VLP surface does not significantly change this rate of uptake (Figure S4, Supporting Information). To assess if the increased rate of uptake of iRGD‐conjugated PhMV is related to changes in the mechanism of uptake, we performed confocal microscopy with nuclear staining using DAPI and cell surface staining using fluorescently labeled wheat‐germ agglutinin (Figure S5, Supporting Information). Whereas PhMV‐Cy5 particles are seen in the endolysosomal compartment, iRGD‐PhMV‐Cy5 particles are clustered in punctate foci adjacent to the plasma membrane (Figure 2E,F).

### 2.4 Preparation and Characterization of PEGylated iRGD‐PhMV Nanoparticles

Our group and others have shown that the addition of poly(ethylene glycol) (PEG) to the surface of viral nanoparticles can reduce immune clearance and improve pharmacokinetics,^[^ ^16^ ^]^ which is consistent with synthetic NP formulations.^[^ ^32^ ^]^ In our previous work with PhMV VLPs, we utilized a 2000 Da PEG (PEG2K) coating and hence we adapted these methods here. We generated iRGD‐conjugated PEG2K‐PhMV NPs by conjugating maleimide‐PEG2K‐NHS esters to the surface lysines of PhMV, and then linking fluorescein–cysteine–aminohexanoic acid–iRGD (FAM‐Cys‐iRGD) using thiol‐maleimide chemistry (Figure S6A, Supporting Information). A PEGylated iRGD‐free control, PEG2K‐PhMV‐Cy5, was also synthesized. Again, the final product, iRGD‐PEG2K‐PhMV‐Cy5 remained structurally sound (Figure S6B–G, Supporting Information). Based on gel migration by SDS‐PAGE, approximately half of all PhMV CPs were conjugated to PEG2K, corresponding to approximately 90 PEG2K molecules per VLP. Based on absorbance measurements, ≈17 FAM‐Cys‐Ahx‐iRGD were conjugated per VLP. iRGD‐PEG2K‐PhMV‐Cy5 binds αvβ3 integrin with affinities similar to that of iRGD‐PhMV‐Cy5, indicating preserved structure and function of surface‐bound iRGD peptides (Figure S7A,B, Supporting Information).

### 2.5 In vivo Biodistribution of iRGD‐PhMV Nanoparticles

**Figure 3:**
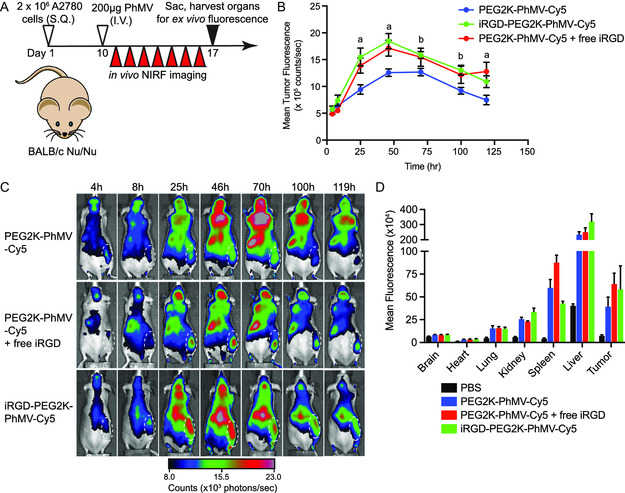
Conjugation or coadministration of iRGD increases uptake of PhMV in the A2780 xenograft model. A) A2780 cells were injected subcutaneously into BALB/c Nu/Nu mice on day 1 followed by intravenous administration of PBS, PEG2K‐PhMV‐Cy5, PEG2K‐PhMV‐Cy5 + 4 μmol kg^−1^ iRGD peptide, or iRGD‐PEG2K‐PhMV‐Cy5 on day 10. Mice are imaged daily using in vivo NIRF imaging for 7 days, then euthanized and organs are analyzed for ex vivo fluorescence. B,C) Quantified in vivo fluorescence of mice treated with Cy5‐PhMV (B) and representative images (C). D) Ex vivo fluorescence of mouse organs and tumor. The error bars represent S.E.M. statistical analysis by two‐tailed *t*‐test (ns: not significant, a: *p* < 0.05 for iRGD‐PEG2K‐PhMV‐Cy5 and PEG2K‐PhMV‐Cy5 + free iRGD, b: *p* < 0.05 for iRGD‐PEG2K‐PhMV‐Cy5).

To assess whether iRGD‐conjugated PhMV NPs home to tumors in vivo, we next analyzed the biodistribution of iRGD‐PEG2K‐PhMV‐Cy5 using an A2780 xenograft tumor model in Nu/Nu BALB/c mice (**Figure** **3**A). After tumors were established, 200 μg of iRGD‐PEG2K‐PhMV‐Cy5 was injected intravenously through the tail vein. Since it has been previously shown that coadministration of iRGD peptide with small molecules and inorganic nanoparticles can induce a similar increase in tumor uptake as compared to direct iRGD peptide conjugation (the so‐called “bystander effect”),^[^ ^26^ ^]^ 200 μg of PEG2K‐PhMV‐Cy5 was also coadministered with 4 μmol kg^−1^ free iRGD peptide. PEG2K‐PhMV‐Cy5 was used as a control. In vivo NIR fluorescence was measured 4 and 8 h after nanoparticle injection, then daily for 7 days. After administration, intratumoral NIR fluorescence peaked after 48–72 h (Figure 3B). There was a 64%, 47%, and 25% increase in intratumoral fluorescence in iRGD‐PEG2K‐PhMV‐Cy5 treated mice after 1, 2, and 3 days, respectively, as compared to control, as well as a 47%, 36%, and 22% increase with iRGD coadministration as compared to PhMV lacking conjugated or coadministered iRGD, indicated increased tumoral PhMV nanoparticle uptake in these conditions. Representative in vivo NIRF images show there is also significant nonspecific nanoparticle uptake in the liver and spleen (Figure 3C), with complete data in supplemental Figure S8, Supporting Information. Six days after injection, the animals were euthanized and ex vivo NIRF analysis of the tumors and major organs was performed. There is a trend toward, but no statistically significant difference between ex vivo tumor NIRF signals at this timepoint. There is no statistically significant difference in splenic or hepatic uptake between iRGD‐conjugated PhMV nanoparticles and nontargeted controls (Figure 3D, S8B–E, Supporting Information). Overall data show tumor homing of the VLPs with increased tumor accumulation of iRGD formulations either by conjugation to or coadministration with the VLPs.

### 2.6 Preparation and Characterization of Doxorubicin‐Loaded iRGD‐PhMV Nanoparticles

**Figure 4:**
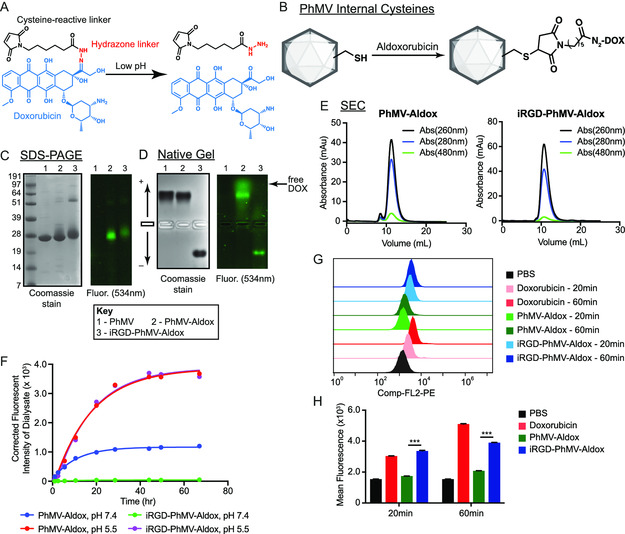
Synthesis and characterization of aldoxorubicin‐iRGD‐PhMV conjugates. A) Chemical components of aldoxorubicin. B) Scheme for conjugation of aldoxorubicin to internal cysteines of PhMV. C–E) Characterization of iRGD‐PhMV‐Aldox and PhMV‐Aldox using SDS‐PAGE (C), native gel electrophoresis (D), and size exclusion chromatography (E). F) Fluorescence intensity of dialysate after dialysis of PhMV‐Aldox or iRGD‐PhMV‐Aldox into PBS (pH 7.4) or 50 mm Na citrate (pH 5.5) at 37 °C. G,H) iRGD‐PhMV‐Aldox and PhMV‐Aldox uptake by A2780 cells after 20 and 60 min, as measured by flow cytometry (G) with quantification (H). The error bars represent S.E.M. of three replicates. Statistical analysis by two‐tailed *t*‐test (***: *p* < 0.001).

We have previously shown that the chemotherapeutic doxorubicin can be conjugated to PhMV nanoparticles through thiol‐maleimide chemistry using its derivative aldoxorubicin (**Figure** **4**A).^[^ ^33^ ^]^ The hydrazone linker of aldoxorubicin is designed to release free doxorubicin within the acidic tumor microenvironment and/or the endolysosomal compartment of tumor cells.^[^ ^34^ ^]^ PhMV was loaded with aldoxorubicin through reaction with its internal cysteines, then coupled to iRGD peptides to generate iRGD‐PhMV‐Aldox (Figure 4B). The final product was not substantially altered in its nanostructure as compared to PhMV VLPs or other iRGD‐PhMV nanoparticles and was monodisperse and homogenous (Figure 4C–E, S9A, Supporting Information). Based on UV–vis spectroscopy, ≈70 doxorubicin molecules were conjugated per iRGD‐PhMV‐Aldox NP (Figure S9B,C, Supporting Information). Owing to the labile nature of the hydrazone linker, a small amount of free doxorubicin was seen in some characterization methods and likely represents doxorubicin that is noncovalently contained within the PhMV nanoparticles, likely due to supramolecular π–π stacking of doxorubicin.

### 2.7 In vitro Drug Release and Cytotoxicity of iRGD‐PhMV‐Aldox Nanoparticles

We next assessed if aldoxorubicin‐conjugated PhMV nanoparticles functioned as expected in vitro. We observed that free doxorubicin is rapidly released from iRGD‐PhMV‐Aldox nanoparticles at low pH (pH 5.5) but not at physiologic pH (Figure 4F), which indicates the hydrazone linker functions as expected. To assess if iRGD‐PhMV‐Aldox nanoparticles display increased cellular uptake, we performed flow cytometry. A2780 cells treated with iRGD‐PhMV‐Aldox showed a nine‐ and fourfold increase in internal fluorescence after 20 and 60 min, respectively, as compared to cells treated with PhMV‐Aldox, indicative of increased cellular uptake of iRGD‐PhMV‐Aldox NPs (Figure 4G). The level of cellular uptake of iRGD‐PhMV‐Aldox was similar to that of free doxorubicin (Figure 4H). When treated with a similar absolute dose of doxorubicin, no significant difference in cytotoxicity is observed between iRGD‐PhMV‐Aldox and PhMV‐Aldox, likely due to the long timescale of this assay (72 h) (Figure S10, Supporting Information).

### 2.8 In vivo Efficacy of iRGD‐PhMV‐Aldox in Murine Cancer Model

**Figure 5:**
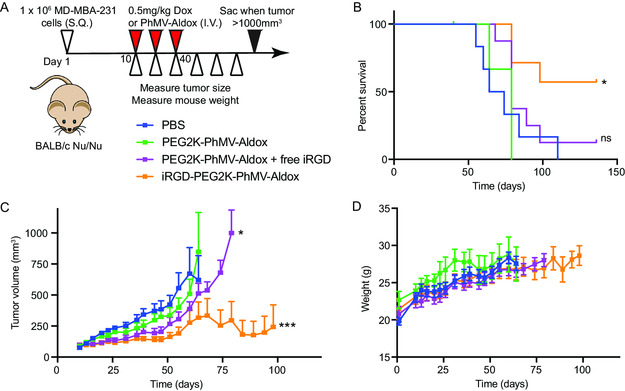
Inhibition of growth in an MDA‐MB‐231 xenograft model. A) MDA‐MD‐231 cells are injected subcutaneously into BALB/c Nu/Nu mice. Treatment began when tumors reached a volume of ≈100 mm^3^ and involved twice‐weekly intravenous bolus of PBS, 0.5 mg kg^−1^ doxorubicin, PEG2K‐PhMV‐Aldox, PEG2K‐PhMV‐Aldox + free iRGD (8 μmol kg^−1^), or iRGD‐PEG2K‐PhMV‐Aldox. Treatment was stopped at day 40. B) Animals were euthanized when tumor volumes reached 1000 mm^3^. Statistical analysis of survival curves was carried out by log‐rank (Mantel–Cox) test, ns: *p* > 0.05, *: *p* < 0.05, as compared to PEG2K‐PhMV‐Aldox. C,D) Mean tumor volumes (C) and mean animal weights (D). The error bars represent the S.E.M. Statistical analysis was carried out by two‐way analysis of variance (ANOVA) (*: *p* < 0.05, ***: *p* < 0.001, as compared to PEG2K‐PhMV‐Aldox).

To assess if iRGD‐PhMV‐Aldox nanoparticles show improved antitumor efficacy, we generated a PEG2Kylated version of these particles for in vivo studies (Figure S11, Supporting Information). We then used a previously established MDA‐MD‐231 xenograft tumor model in Nu/Nu BALB/c mice. Once tumor volume reached ≈100 mm^3^, mice were treated twice weekly with iRGD‐PEG2K‐PhMV‐Aldox, PEG2K‐PhMV‐Aldox coadministered with 8 μmol kg^−1^ free iRGD peptide, or PEG2K‐PhMV‐Aldox (**Figure** **5**A). Nanoparticle dosing was normalized to deliver 0.5 mg doxorubicin per kg animal body weight. In the control (PBS) and PhMV‐Aldox treatment arms, tumor volumes reached 1000 mm^3^ after approximately 50–75 days (Figure S12A,B, Supporting Information). Treatment with iRGD‐PhMV‐Aldox significantly reduced tumor volume and increased survival as compared to control (Figure 5B,C). In fact, four animals showed complete tumor regression at day 136 (Figure S12C, Supporting Information). Coadministration of PEG2K‐PhMV‐Aldox with free iRGD peptide significantly reduced tumor volume but did not significantly alter survival (Figure S12D, Supporting Information). Mouse weights were similar across all treatment groups (Figure 5D).

## 3 Discussion and Conclusion

We have developed a novel iRGD‐peptide conjugated virus‐like nanoparticle for targeted cancer cell uptake. After identifying a strategy for the robust bioconjugation of iRGD peptides to PhMV nanoparticles (Figure 1), we show that iRGD‐PhMV is rapidly taken up by cultured cancer cells in vitro (Figure 2) and has increased intratumoral localization in vivo (Figure 3). We then show that iRGD‐PhMV can be loaded with the cytotoxic drug doxorubicin (Figure 4) and show that iRGD‐PhMV‐Aldox shows improved antitumoral efficacy as compared to nontargeted control (Figure 5).

By using click chemistry with propargylglycine‐iRGD peptides and azide‐functionalized PhMV, we were able to vary the number of surface‐bound iRGD peptides on iRGD‐PhMV NPs. A fourfold change in the stoichiometry of iRGD peptide used during conjugation led to a nearly 20‐fold increase in the αv integrin binding affinity of iRGD‐PhMV NPs (Figure 2A,B). However, increasing the biochemical affinity of multivalent nanoparticles for their target does not directly increase on‐target binding or specificity since increased ligands on NP surfaces can reduce particle circulatory time and alter the mechanics of target engagement.^[^ ^35^ ^]^ In fact, a reduction in the density of anti‐intercellular adhesion molecule‐1 (ICAM‐1) antibodies on the surface of poly(4‐vinylphenol) (PVPh) NPs was found to paradoxically increase the specificity of target engagement.^[^ ^36^ ^]^ This suggests that further characterization of iRGD‐VLPs may be needed to empirically determine the appropriate surface iRGD‐peptide concentration to maximize tumor‐homing properties.

Since plant viruses have not evolved to be infectious toward mammalian cells, plant viral NPs and VLPs typically enter the cytoplasm of mammalian cells through passive endocytosis^[^ ^37^ ^]^ or interaction with highly evolutionarily conserved surface proteins, such as the interaction of cowpea mosaic virus (CPMV) and other members of the picornavirus superfamily with vimentin.^[^ ^38^, ^39^ ^]^ The rate of uptake of iRGD‐PhMV that we observed in this study is significantly higher than nonliganded PhMV (Figure 2C,D) and is approaching the rate of cellular entry for the small molecule doxorubicin (Figure 4G,H). This rate of cellular internalization, along with the punctate foci of iRGD‐PhMV‐Cy5 observed by confocal microscopy (Figure 2E), suggests active cellular uptake of iRGD‐PhMV NPs. This may be due to αv integrin‐mediated cellular uptake or activation of NRP‐1‐mediated micropinocytosis of the C‐end rule (CendR) peptide that is revealed after proteolytic cleavage of iRGD‐peptides.^[^ ^25^, ^40^ ^]^

The similar degree of tumor localization of iRGD‐PEG2K‐PhMV‐Cy5 and PEGK2K‐PhMV‐Cy5 coadministered with free iRGD peptide is consistent with prior work that showed similar tumor accumulation of 130 nm nanoparticles consisting of albumin‐embedded paclitaxel after conjugation to or coadministration with iRGD‐peptide.^[^ ^26^ ^]^ However, we still observed significant nonspecific uptake of PhMV NPs in the liver and spleen, despite surface shielding with PEG. The trend toward increased uptake of iRGD‐PhMV NPs observed in the liver and kidney may be due to the native expression of *ITGA3* and *ITGA5*, which encode for β3 and β5 integrin subunits, in endothelial cells of these organs.^[^ ^41^ ^]^ Further optimization of surface iRGD peptide concentration may provide a strategy to reduce these nontumoral binding events, although it is likely that these will remain sites of nonspecific uptake for all PhMV VLPs despite surface peptide targeting. Although hepatotoxicity has not been significantly observed in clinical trials of doxorubicin nanoparticles^[^ ^42^ ^]^ or preclinical testing of doxorubicin‐containing VLPs,^[^ ^43^ ^]^ further work and efficacy studies of iRGD‐PhMV‐Aldox will be critical to ensure safety.

Since the tumoral delivery of PhMV‐Cy5 was increased by nearly 50% with iRGD‐peptide coadministration or conjugation after 48 h and because of the long tumoral residency time of PhMV VLPs (Figure 3B), we hypothesized that multiple repeat administrations of iRGD‐conjugated PhMV‐Aldox would result in a multiplicative increase in the delivery of doxorubicin over time. As evidence of this, twice weekly dosing of 0.5 mg kg^−1^ doxorubicin by iRGD‐PEG2K‐PhMV‐Aldox particles is significantly more effective than delivery of the same dose by nontargeted PEG2K‐PhMV‐Aldox particles (Figure 5). In fact, a complete response was observed in 4/7 animal treated with iRGD‐PEG2K‐PhMV‐Aldox while none were observed with PEG2K‐PhMV‐Aldox treatment. The reduced effect of coadministration of iRGD‐peptide with PEGK2K‐PhMV‐Aldox as compared to direct conjugation in this setting may be due to a mismatch between the timing of the “bystander effect,” which peaks 15–30 min after iRGD‐peptide administration^[^ ^44^ ^]^ and the long serum half‐life of PhMV VLPs.^[^ ^19^ ^]^ Additionally, there could be altered transit of iRGD‐conjugated versus coadministered PhMV VLPs into the tumor parenchyma. The low efficacy of nontargeted PEG2K‐PhMV‐Aldox is likely due to the low dose of doxorubicin administered (0.5 mg kg^−1^) compared to the recommended clinical dose of 60–75 mg m^−2^ or approximately 1.5 mg kg^−1^ in breast cancer combination therapy (Lexicomp, Inc.). That a lower cumulative dose of doxorubicin is still efficacious when delivered as an iRGD peptide‐targeted nanoparticle suggests this method of drug deliver could be used to reduce systemic toxicity of doxorubicin and perhaps other chemotherapeutics while maintaining on‐target potency.

Several prior studies have employed doxorubicin‐loaded VLPs for targeted tumor delivery. The direct intratumoral delivery of tobacco mosaic virus (TMV) disks conjugated to aldoxorubicin showed increased efficacy in an intracranial xenograft model of glioblastoma as compared to free doxorubicin^[^ ^45^ ^]^ and our group has previously demonstrated that nontargeted PEG2K‐PhMV‐Aldox VLPs display increased efficacy as compared free doxorubicin.^[^ ^33^ ^]^ This work differs from these prior studies in the use of iRGD peptide‐based targeting. Additionally, a prior study used genetic engineering to insert RGD peptides into the major immunodominant loop region (MIR) of hepatitis B core protein (HBc) VLPs and used disassembly–reassembly to encapsulate doxorubicin into the VLP.^[^ ^43^ ^]^ Our work differs from this study in two regards. First, we use acid‐labile conjugation of doxorubicin to PhMV VLPs, which adds an additional layer of specificity of tumoral delivery. Second, we use nongenetic strategies for RGD peptide–VLP conjugation, which allows for facile alterations in peptide:VLP stoichiometry, as demonstrated in Figure S2–S4, Supporting Information, as well as the use of the more advanced iRGD peptide, which takes additional advantage of CendR‐mediated tumoral uptake. Taken together, these data suggest that iRGD‐PhMV VLPs hold high promise for the targeted delivery of chemotherapeutics and other small molecules to tumors after intravascular injection.

## 4 Experimental Section

4.1

4.1.1

##### Preparation of PhMV VLPs

PhMV VLPs were prepared by expressing the coat protein in BL21(DE3) as previously described.^[^ ^18^ ^]^ Briefly, BL21(DE3) was transformed with pRSETa‐PhMV CP. A single colony was isolated and used to inoculate 50 mL of Luria Broth (LB, Sigma) supplemented with carbenicillin at 50 μg mL^−1^, and grown overnight at 37 °C. This was used to inoculate 1 L of terrific broth (TB, Sigma Aldrich) supplemented with carbenicillin at a 1:100 dilution. Cultures were grown at 37 °C to OD_600_ ≈1.0 and induced with 0.5 mm IPTG (Sigma) at 30 °C overnight. Cultures were then pelleted, lysed by sonication in 50 mm sodium citrate pH 5.5 (SCB), and clarified at 30 000 × g for 30 min at 4 °C. VLPs were precipitated using 10% w/v polyethylene glycol (PEG), resuspended in SCB, then purified by ultracentrifugation using a 50.2 Ti rotor at 35 000 rpm for 3 h at 4 °C. Pellets were resuspended overnight in SCB then layered onto a 10%–40% linear sucrose gradient and separated using a SW32 rotor at 28 000 rpm for 3 h at 4 °C. The light scattering zone was collected, diluted with SCB, and centrifuged at 42 000 rpm for 3 h at 4 °C using a 50.2 Ti rotor. The final pellet was resuspended in SCB to yield the pure VLP, which was stored at 4 °C. Protein concentration was determined by BCA Assay (Thermo Fisher) using BSA as a standard.

##### Bioconjugation Reactions

Internal cysteine residues of PhMV particles (at 1.5 mg mL^−1^) in 10 mm potassium phosphate (KP) pH 7.5 were alkylated using maleimide‐sulfoCy5 (LumiProbe) at 3 molar equivalents per coat protein (eq/CP) or aldoxorubicin (Aldox, MedChem Express) at 5 eq/CP at room temperature overnight. The resulting product was purified by ultracentrifugation (121, 139*g*, 70 min, 4 °C over a sucrose cushion (30% sucrose)). The pellet was dissolved in 10 mm KP pH 7.5 and used for further bioconjugation reactions. External lysine residues of PhMV particles (at 1.5 mg mL^−1^) in 10 mm KP pH 7.5 were acylated using NHS esters (NHS‐PEG4‐N_3_, NHS‐PEG2K, or NHS‐PEG2K‐maleimide) (Nanocs) at 50 eq/CP for 3 h at room temperature followed by purification by ultracentrifugation as above. Copper‐catalyzed azide–alkyne cycloaddition (CuAAC) reactions were performed using N_3_‐PhMV‐Cy5 or N_3_‐PhMV‐Aldox (at 1 mg mL^−1^) in 10 mm KP pH 7.0; particles were reacted with 1 mm CuSO_4_ (Sigma), 200 μm tris‐hydroxypropyltriazolylmethylamine (THPTA, Click Chemistry Tools), 5 mm aminoguanidine (AMG, Sigma), and 5 mm sodium ascorbate (Sigma) with 1 eq/CP propargylglycine‐aminohexanoic acid‐iRGD (Pra‐iRGD), unless otherwise noted, for 1 h at room temperature and purified by ultracentrifugation, as above (CuSO_4_, THPTA, AMG, and sodium ascorbate were premixed as a 10× master mix, then added to PhMV particles prior to the addition of Pra‐iRGD). Two eq/CP fluorescein‐Cys‐aminohexanoic acid‐iRGD (FAM‐Cys‐iRGD) was conjugated to Mal‐PhMV‐Cy5 or Mal‐PhMV‐Aldox at 1 mg mL^−1^ in 10 mm KP pH 7.5 at room temperature overnight, quenched with 100 eq/CP beta‐mercaptoethanol (Sigma) at room temperature for 1 h, and purified by ultracentrifugation, as above. Final particles were buffer exchanged into PBS, concentrated to >5 mg mL^−1^, passed through a 0.22 μm filter, and stored at 4 °C and used within 2 weeks of synthesis. Concentration was determined by BCA assay (Thermo Fisher) using BSA as a standard.

##### Particle Characterization

Final particles were characterized by SDS‐PAGE (12% Bis‐Tris; Novex, Thermo Fisher), native gel electrophoresis (0.8% w/v agarose in TBE), UV–vis (Nanodrop 200 spectrophotometer, Thermo Fisher), size exclusion chromatography (Superose 6 Increase 10/300 GL column at 0.5 mL min^−1^ on a AKTA FPLC, GE), dynamic light scattering (Zetasizer Nano ZSP/Zen5600, Malvern Panalytical), and transmission electron microscopy with 400‐mesh hexagonal copper grids using UAc‐negative‐staining (2% w/v) and a FEI TecnaiSpirit G2 BioTWIN TEM at 80 kV for image acquisition, as applicable. The concentration of PhMV‐bound sulfo‐Cy5, doxorubicin, and FAM‐Cys‐iRGD was determined by UV–vis spectroscopy using the extinction coefficients e(sulfo‐Cy5, 646 nm) = 271 000 m ^−1^ cm^−1^, e(DOX, 488 nm) = 11 500 m ^−1^ cm^−1^, and e(FAM, 495 nm) = 20 960 M^−1^ cm^−1^.

##### Integrin‐Binding ELISA

Ninety‐six well plates (MaxiSorp, Thermo Fisher) were incubated with 0.5 μg mL^−1^ recombinant αvβ3 integrin (R&D Systems) in 100 mm KP pH 8.0 at 4 °C overnight. Plates were then blocked with 5% w/v bovine serum albumin (BSA, Sigma) in PBS supplemented with 1 mm CaCl_2_, 0.5 mm MgCl_2_, and 0.1% v/v Tween‐20 (PBSD‐T). Plates were then incubated for 90 min at room temperature with serial dilutions of PhMV VLPs (25 nm → 1.6 pm) in 2% w/v BSA/PBSD‐T. Plates were then washed with PBSD‐T, incubated with rabbit anti‐PhMV primary antibodies (1:1000, Pacific Immunology) in 5% w/v BSA/PBSD‐T followed by HRP‐conjugated goat antirabbit secondary antibodies (1:5000; Thermo Fisher). Plates were then developed using TMB‐ELISA substrate (Thermo Fisher) and absorbance was measured at 450 nm. All conditions were performed in triplicate. Curves were fit using at least‐squares method (Prism, GraphPad).

##### Flow Cytometry

A2780 cells were grown to approximately 75% confluence in RPMI media (Corning) supplemented with 10% v/v fetal bovine serum (FBS, R&D Systems) and 1% v/v penicillin–streptomycin (Cytiva) at 37 °C in a 5% CO_2_ humidified incubator. Cells were isolated using nonenzymatic cell dissociation buffer (Gibco) and resuspended at 1.0 × 10^7 ^cells mL^−1^ in RPMI. Cells were incubated with 2.5 × 10^6^ particles cell^−1^ for the specified time (5–60 min), washed three times with ice‐cold PBS, then analyzed for internal fluorescence using the APC channel (Cy5 particles) or PE channel (Aldox particles) on an Accuri C6 Plus (BD Biosciences). At least 10 000 live events were acquired per sample, and all conditions were performed in triplicate. Results were analyzed with FlowJo (BD Biosciences) and statistical significant was determined by Student's *t*‐test (Prism, GraphPad).

##### Confocal Microscopy

A2780 cells were plated at 25 000 cells well^−1^ in a 24‐well plate on circular glass coverslips and grown overnight using the above‐growth conditions. PhMV particles were added directly to the media at 2.5 × 10^6^ particles cell^−1^ for 10 min. Cells were then transferred to 4 °C, washed with cold PBS, and fixed with fixation buffer (4% paraformaldehyde, 0.3% glutaraldehyde in PBS) for 5 min at room temperature. Cells were then stained with wheat‐germ agglutinin–Alexa Fluor 488 (WGA; Sigma) in 5% BSA/PBS and mounted on glass slides using Fluoroshield with DAPI (Sigma). Slides were then analyzed on an A1R confocal microscope (Nikon). Image analysis and final images were created using Fiji (NIH).^[^ ^46^ ^]^

##### Doxorubicin Release Assay

PhMV‐Aldox particles at 2 mg mL^−1^ in microdialysis chambers (Thermo Fisher) were incubated in PBS (pH 7.4) or 50 mm sodium citrate (pH 5.5) and doxorubicin release were measured by a plate reader (excitation 488 nm, emission 595 nm), as previously described.^[^ ^33^ ^]^

##### Cytotoxicity Assay

A2780 cells were plated at 3000 cells well^−1^ in 96 well plates and grown overnight using the above‐growth conditions. Serial dilutions (20 μm → 1.3 nm) of doxorubicin or PhMV‐Aldox particles (normalized to doxorubicin concentration) were added and plates were incubated for 72 h. Viable cells were then analyzed using a CellTiter Glo assay (Promega) according to the manufacturer's protocol. All conditions were performed in triplicate. Curves were fit using a least squares methods (Prism, GraphPad).

##### In Vivo Biodistribution Study

All animal experiments were carried out according to IACUC‐approved procedures at the University of California, San Diego. Mice were anesthetized for all procedures (2.5% isoflurane, O_2_ flow 2.0 L min^−1^). Mice were maintained on an alfalfa‐free (low fluorescence) diet (2018S Teklad, Envigo). A2780 cells were grown to 75% confluence using the above growth conditions, harvested using Trypsin‐EDTA (Corning), and injected subcutaneously at a concentration of 2 × 10^6^ cells mL^−1^ in a 1:1 mixture of RPMI:Matrigel (Corning) into the flank of female BALB/c Nu/Nu mice at 4–6 weeks of age. After 10 days, tumor‐bearing mice were allocated into one of four treatment groups (PBS, PEG2K‐PhMV‐Cy5, PEG2K‐PhMV‐Cy5 + 4 μmol kg^−1^ iRGD, or iRGD‐PEG2K‐PhMV‐Cy5; *n* = 5 per group) and intravenously dosed with 200 μg PhMV particles in 100 μL PBS via tail vein. For in vivo NIRF imaging, mice were scanned at approximately 4, 8, 24, 48, 72, 96, and 120 h after injection using an IVIS 200 small‐animal imaging system (Xenogen, using Cy5.5 excitation and emission filters). After the final NIRF imaging, mice were euthanized and the tumor and major organs were removed and imaged for ex vivo fluorescence. The tissues were then weighed and homogenized and analyzed for fluorescence using a plate reader (Tecan).

##### In Vivo Treatment Study

MDA‐MB‐231 cells were grown in DMEM (Corning) supplemented with 10% FBS and 1% pen/strep, harvested using Trypsin‐EDTA (Corning), and injected subcutaneously at a concentration of 1 × 10^6^ cells mL^−1^ in a 1:1 mixture of RPMI:Matrigel (Corning) into the flank of female BALB/c Nu/Nu mice at 4–6 weeks of age, as previously described.^[^ ^33^ ^]^ Tumor‐bearing mice were allocated into one of four treatment groups (PBS, PEG2K‐PhMV‐Aldox, PEG2K‐PhMV‐Aldox + 8 μmol kg^−1^ iRGD, or iRGD‐PEG2K‐PhMV‐Aldox) when tumor volume reached ≈100 mm^3^. The mice were intravenously injected twice per week with the appropriate treatment at a dosage of 0.5 mg doxorubicin per kg body weight. Treatments were stopped after nine doses (39 days postinjection). Tumor size and body weight were measured before each injection and twice weekly afterward, and total tumor volume was calculated using the formula *V* = l × *w* ^2^/2. Mice were euthanized when tumor volume reached 1000 mm^3^, according to IACUC guidelines.

## Conflict of Interest

N.F.S. is a co‐founder of, has equity in, and has a financial interest with Mosaic ImmunoEnginering Inc. N.F.S. serves as Director, Board Member, and Acting Chief Scientific Officer, and paid consultant to Mosaic. The other authors declare no potential conflict of interest.

## Author Contributions

K.J.B. and Z.Z.: performed experiments. K.J.B. and N.F.S.: designed and analyzed experiments. K.J.B. and N.F.S.: wrote the manuscript.

## Supporting information

Supplementary Material

## Data Availability

The data that support the findings of this study are available from the corresponding author upon reasonable request.
