# Supplementary material for: iRGD‐Targeted Physalis Mottle Virus Like Nanoparticles for Targeted Cancer Delivery
Source: Small Sci. 2023 Jun 27;3(8):2300067. doi: 10.1002/smsc.202300067 (PMC10923535; doi:10.1002/smsc.202300067)
Supplement: Supplementary file 1 — Supplementary Material [file SMSC-3-2300067-s001.pdf]

Supporting Information for

iRGD-targeted Physalis Mottle Virus-like Nanoparticles for Targeted Cancer Delivery

Krister J. Barkovich[1], Zhongchao Zhao[2,3], Nicole F. Steinmetz[1,2,3,4,5,6,7*]*

[1] *Department of Radiology, University of California, San Diego, San Diego, CA*
[2] *Department of NanoEngineering, University of California, San Diego, San Diego, CA*
[3] *Center for Nano-ImmunoEngineering, University of California, San Diego, San Diego, CA*
[4] *Department of Bioengineering, University of California, San Diego, San Diego, CA*
[5] *Institute for Materials Discovery and Design, University of California, San Diego, CA*
[6] *Moores Cancer Center, University of California, San Diego, San Diego, CA*
[7] *Center for Engineering in Cancer, Institute for Engineering in Medicine, University of California, San Diego, San Diego, CA*

**co-corresponding: kbarkovich@health.ucsd.edu and nsteinmetz@ucsd.edu*

Supplemental Figure 1

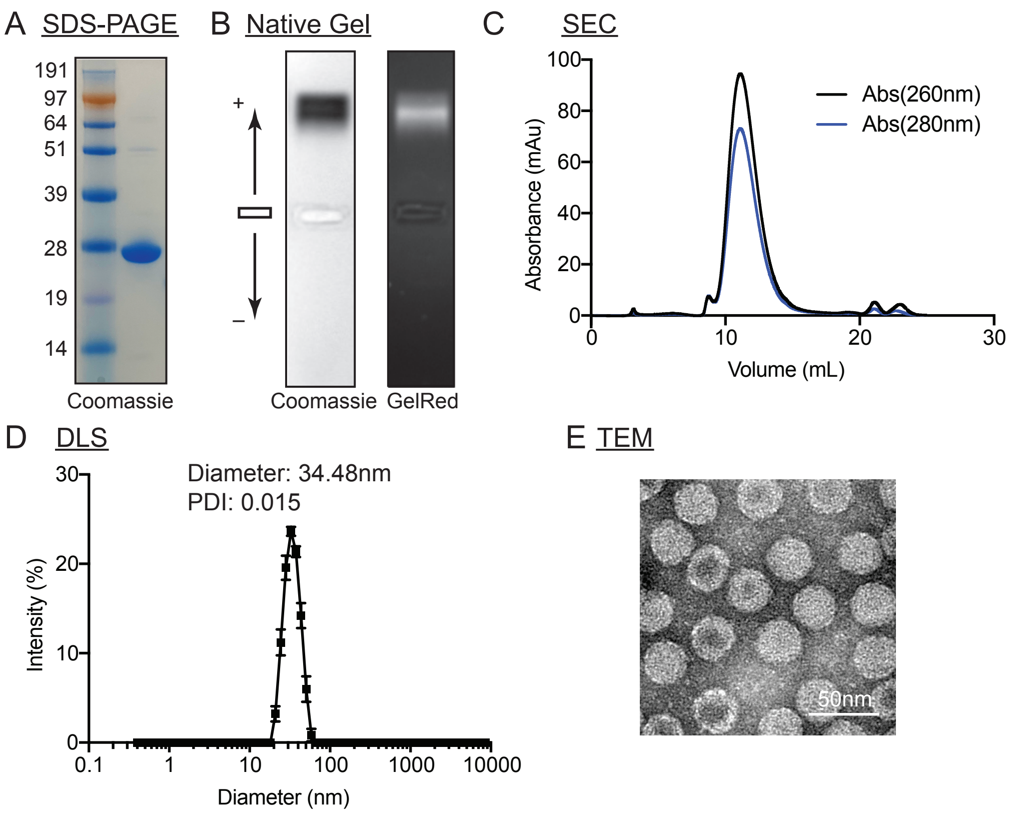

Supplemental Figure 1. Purification of PhMV-like nanoparticles (PhMV). Characterization of purified PhMV by SDS-PAGE (A), native gel electrophoresis (B), size-exclusion chromatography (C), dynamic-light scattering (D), and transmission electron microscopy (E).

Supplemental Figure 2

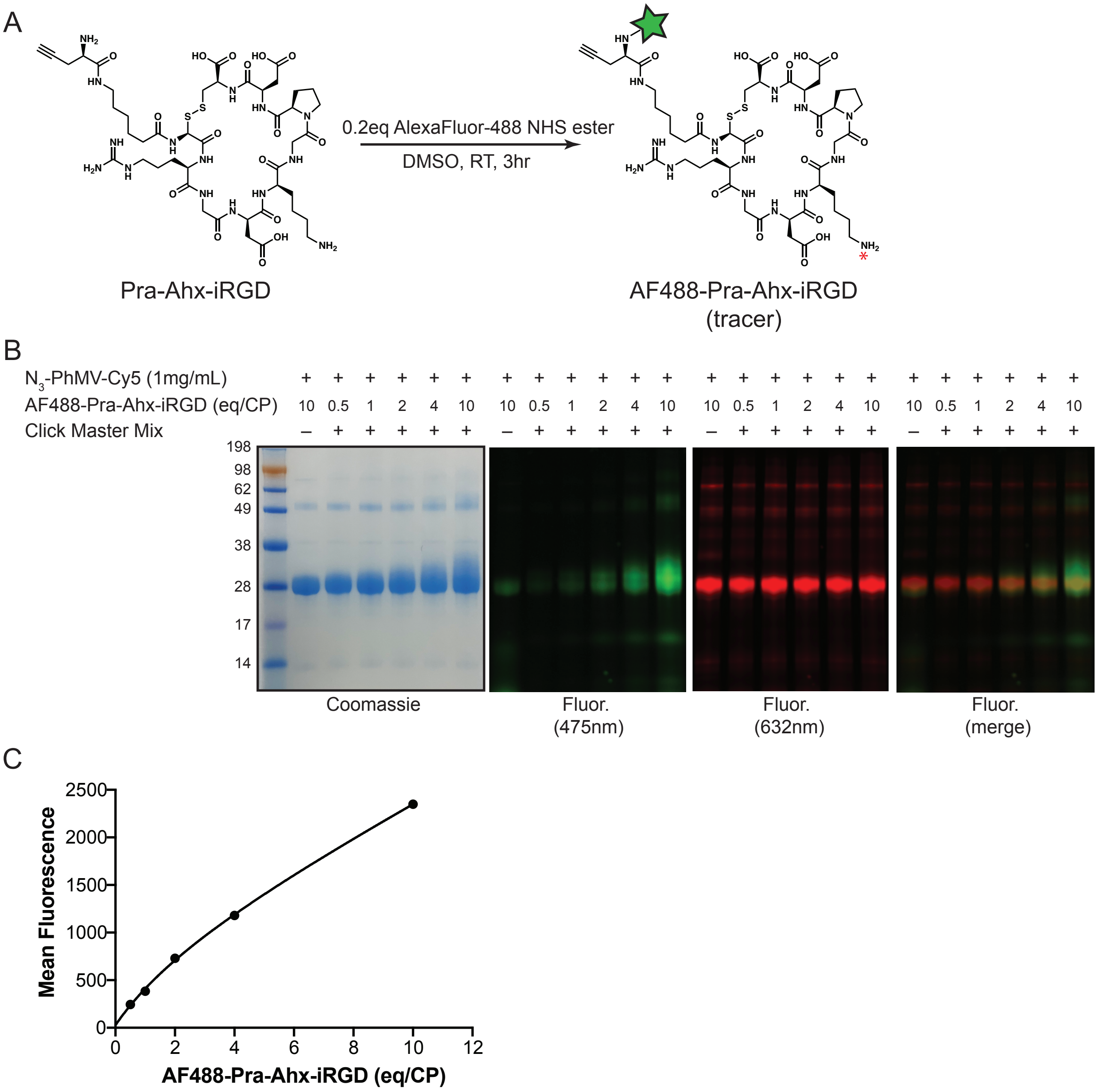

Supplemental Figure 2. Bioconjugation of iRGD peptide to PhMV. A. The tracer AlexaFluor-488-labeled Propargylglycine-aminohexaoic acid-iRGD peptide (AF488-Pra-Ahx-iRGD) was synthesized by reacting Pra-Ahx-iRGD with 0.2 molar equivalents AF-488 NHS ester in DMSO at room temperature for 3 hours. The crude material was used without purification. B. Test copper(I)-catalyzed azide-alkyne cycloaddition (CuAAC) reactions with 0.5 – 10 eq/CP crude AF488-Pra-Ahx-iRGD were performed with N3-PhMV-Cy5 at room temperature for 1hr and analyzed by SDS-PAGE and in gel fluorescence. C. The fluorescent signal of each band at 475 nm was determined by densitometry and graphed as a function of molar ratio per coat protein (eq/CP).

Supplemental Figure 3

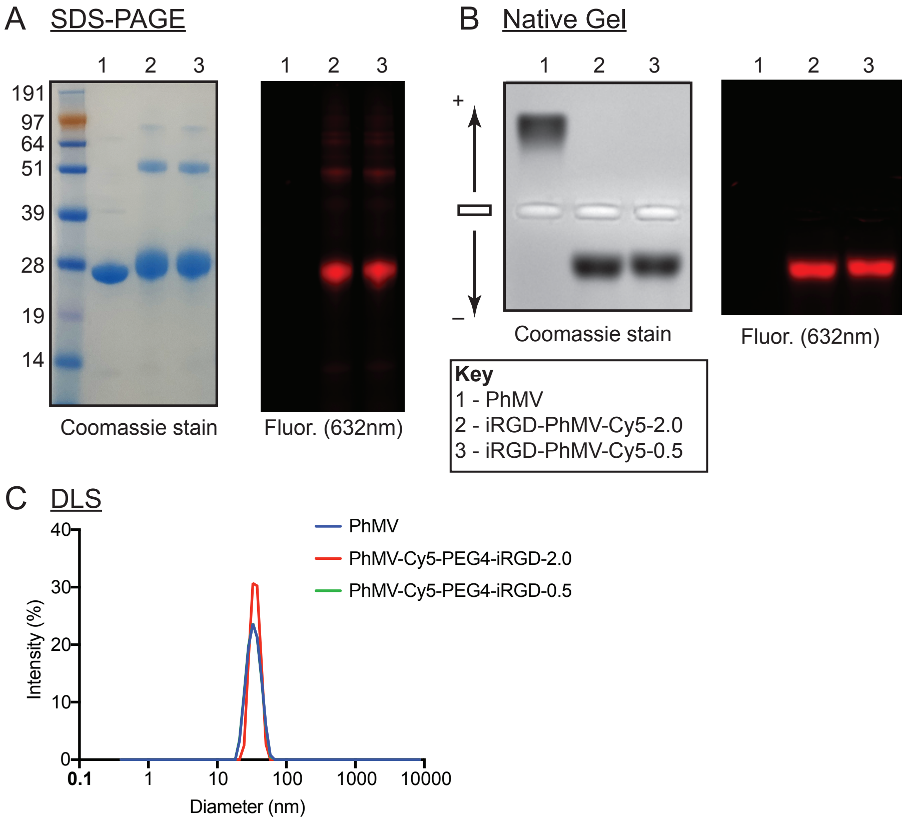

VLP	Hydrodynamic diameter (95% CI)
PhMV	31.64nm (31.45 - 31.82)
iRGD-PhMV-Cy5-2.0	32.87nm (32.72 - 33.01)
iRGD-PhMV-Cy5-0.5	31.44nm (31.30 - 31.59)

Supplemental Figure 3. Purification of iRGD-PhMV-Cy5 panel. Characterization of iRGD-PhMV-Cy5 panel with variable iRGD peptide concentration (0.5 vs 2.0 eq peptide per PhMV coat protein) by SDS-PAGE (A), native gel electrophoresis (B), and dynamic-light scattering (C).

Supplemental Figure 4

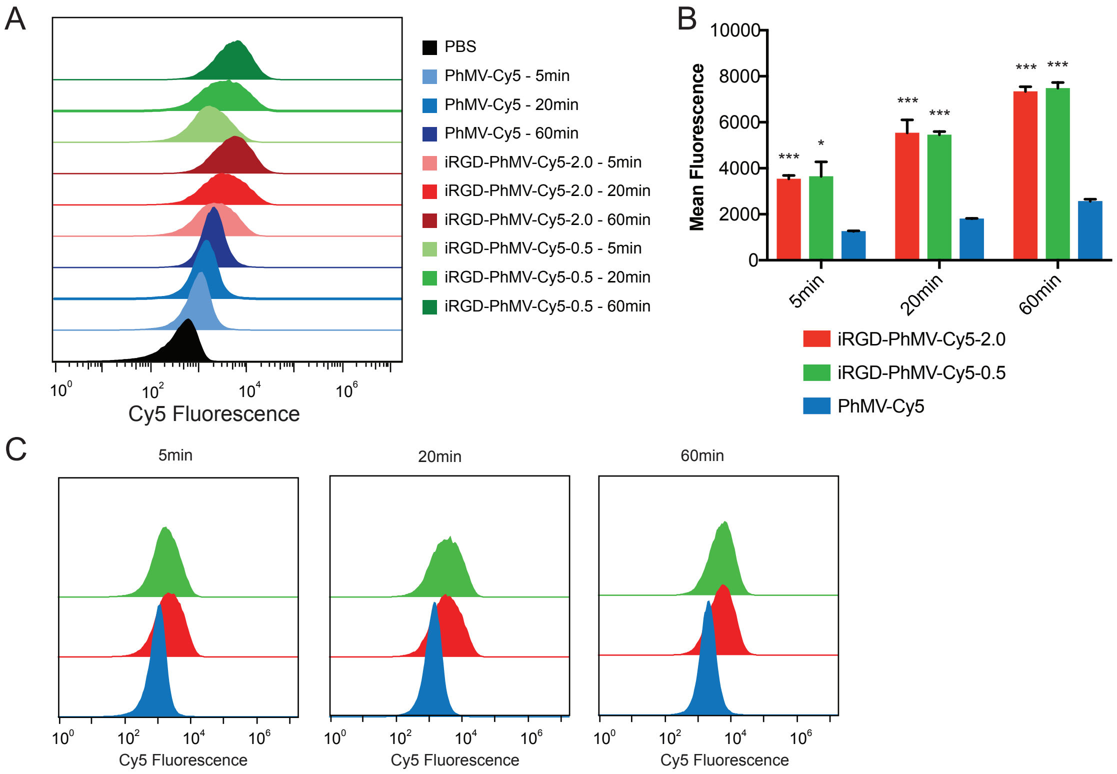

Supplemental Figure 4. iRGD-PhMV is rapidly taken up by A2780 cells. A. iRGD-PhMV-Cy5 panel uptake by A2780 cells after 5, 20, and 60 minutes, as measured by flow cytometry (A) with quantification of three replicates (B), and individual analysis of 5, 20, and 60 minute timepoints (C). Error bars represent the S.E.M. Statistical analysis by two-tailed t-test (*: $p<0.05$, **: $p<0.01$, ***: $p<0.001$, as compared to PhMV-Cy5).

Supplemental Figure 5

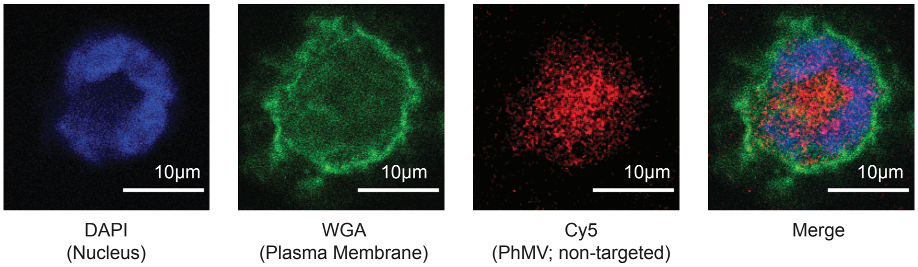

Supplemental Figure 5. Staining scheme for confocal microscopy. A2780 cells analyzed by confocal microscopy are stained with DAPI (nucleus) and wheat germ agglutinin-Alexa Fluor-488 (WGA; plasma membrane). Single color images are acquired separately and stacked into RGB images.

Supplemental Figure 6

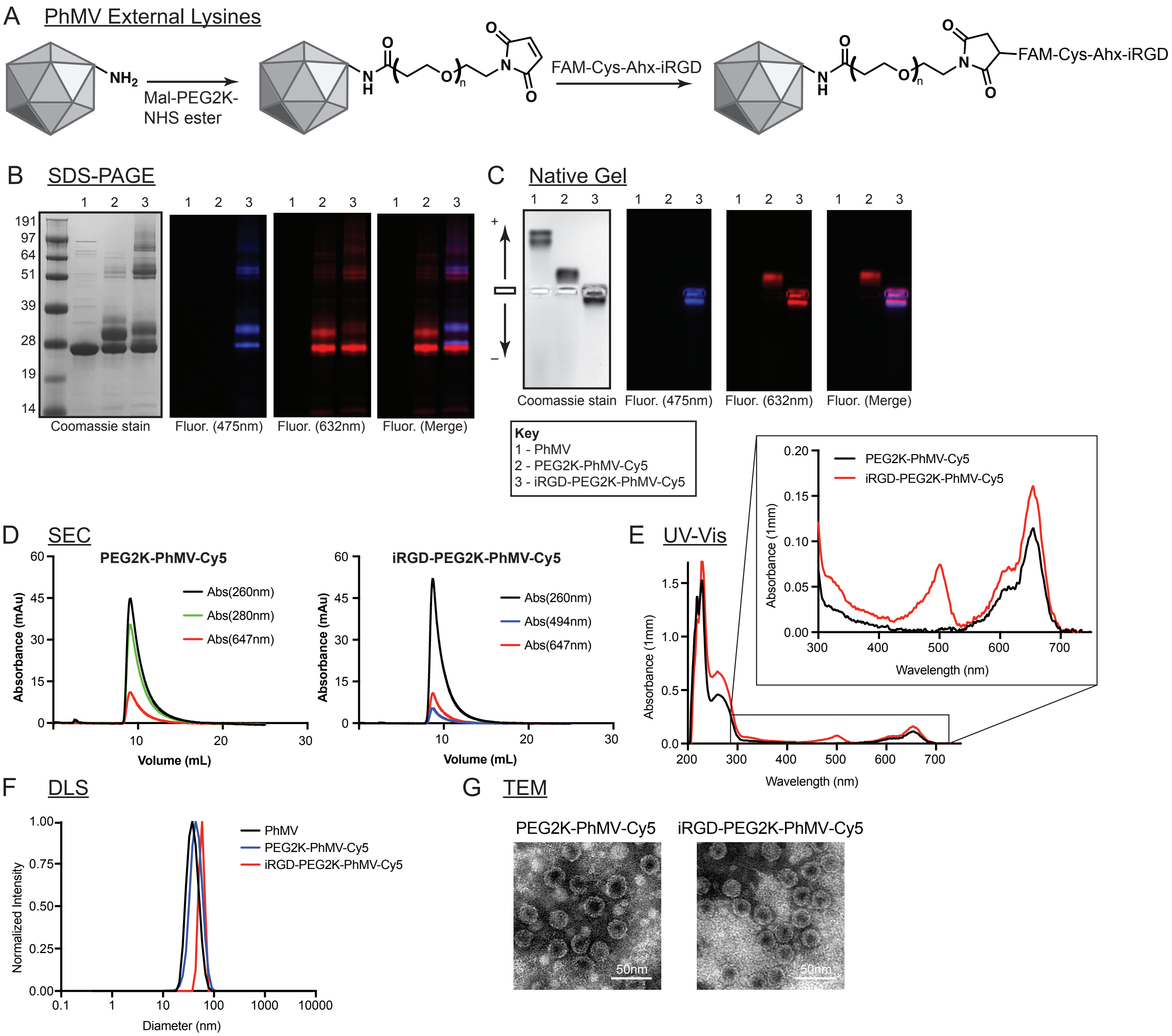

VLP	Hydrodynamic diameter (95% CI)
PhMV	35.86nm (35.46 - 36.26)
PEG2K-PhMV-Cy5	42.00nm (41.71 - 42.30)
iRGD-PEG2K-PhMV-Cy5	53.34nm (52.71 - 53.98)

Supplemental Figure 6. Purification of iRGD-PEG2K-PhMV-Cy5 and PEG2K-PhMV-Cy5. A. Scheme for bioconjugation of Fluorescein-Cys-aminohexanoic acid-iRGD to external lysines of PhMV. B-G. Characterization of PEG2K-PhMV-Cy5 and iRGD-PEG2K-PhMV-Cy5 by SDS-PAGE (B), native gel electrophoresis (C), size-exclusion chromatography (D), UV-Vis spectroscopy (E), dynamic light-scattering F), and transmission electron microscopy (G).

Supplemental Figure 7

A

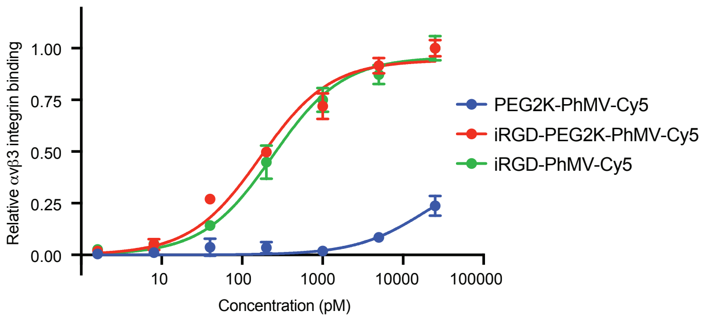

B

PhMV variant	iRGD/CP	K_d (pM, 95% CI)
PhMV-Cy5	0	>25000
iRGD-PhMV-Cy5	1	240.7 (186.0 - 310.6)
iRGD-PEG2K-PhMV-Cy5	1	167.8 (118.0 - 236.8)

Supplemental Figure 7. iRGD-PEG2K-PhMV binds tightly to αvβ3 integrins. A-B. Binding curves (A) and calculated K_d (B) of iRGD-PEG2K-PhMV in αvβ3-binding ELISA.

Supplemental Figure 8

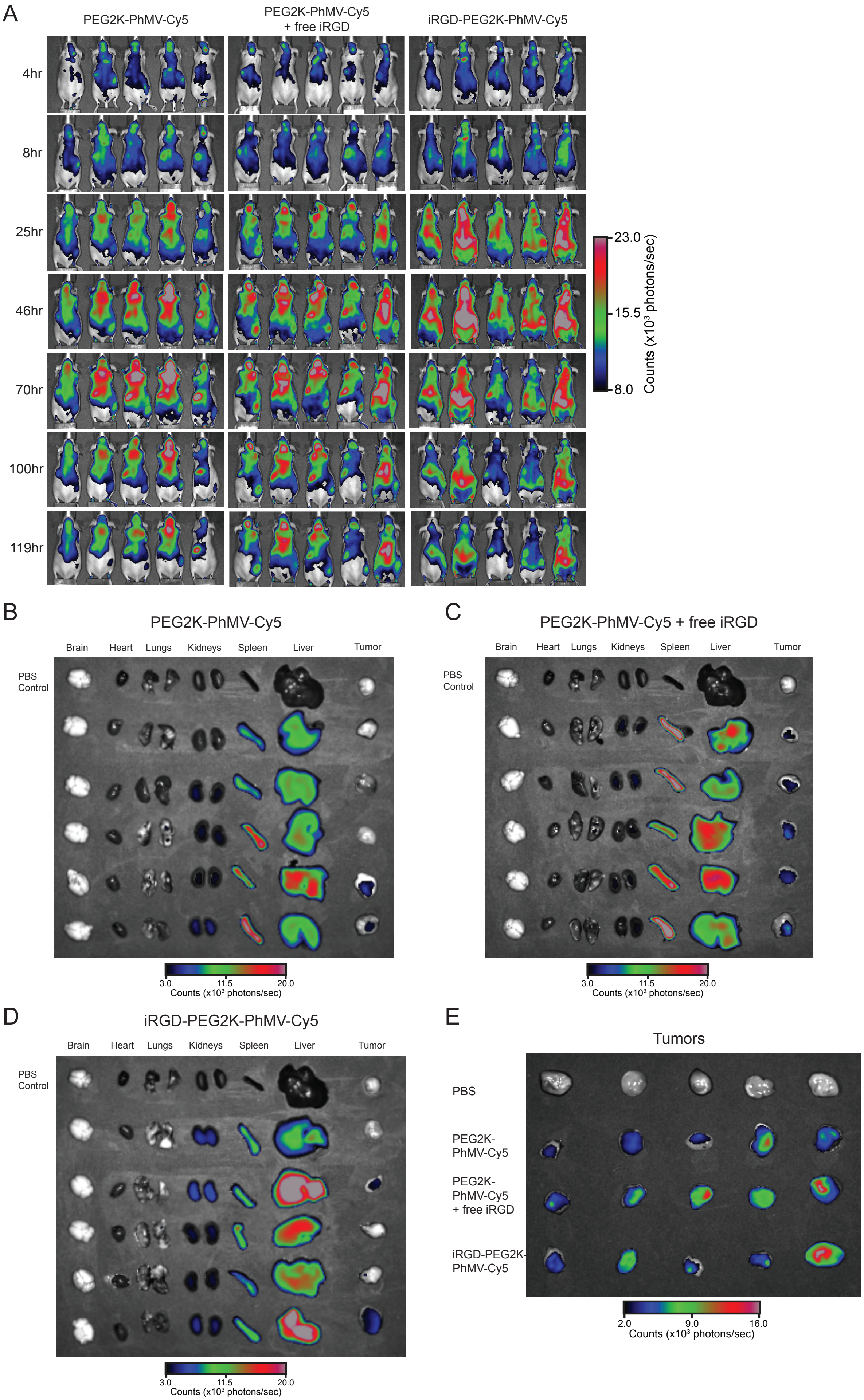

Supplemental Figure 8. Conjugation or co-administration of iRGD increases uptake of PhMV in A2780 xenograft tumor model. A. *In vivo* near IR-fluorescence (NIRF) imaging of mice at selected timepoints for seven days following intravenous injection of PBS, PEG2K-PhMV-Cy5, PEG2K-PhMV-Cy5 + 4mmol/kg iRGD peptide, or iRGD-PEG2K-PhMV-Cy5. B-D. *Ex vivo* NIRF imaging of harvested organs of mice treated with PEG2K-PhMV-Cy5 (B), PEG2K-PhMV-Cy5 + 4mmol/kg iRGD peptide (C), or iRGD-PEG2K-PhMV-Cy5 (D) with representative PBS-control. E. *Ex vivo* NIRF imaging of extracted tumors from all experimental animals.

Supplemental Figure 9

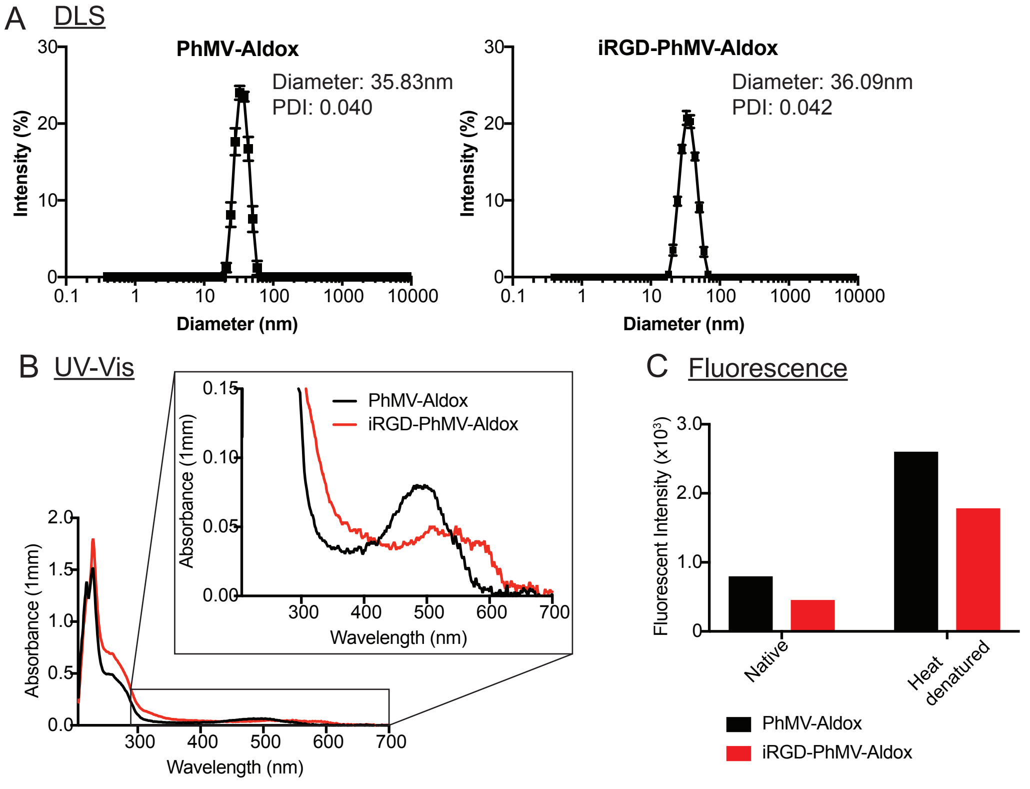

Supplemental Figure 9. Synthesis of Aldoxorubicin-iRGD-PhMV conjugates. A-B. Characterization of PhMV-Aldox and iRGD-PhMV-Aldox by dynamic light scattering (A) and UV-Vis spectroscopy (B). C. Fluorescence of PhMV-Aldox and iRGD-PhMV-Aldox before and after heat denaturation. Error bars represent the S.E.M. of three replicates.

Supplemental Figure 10

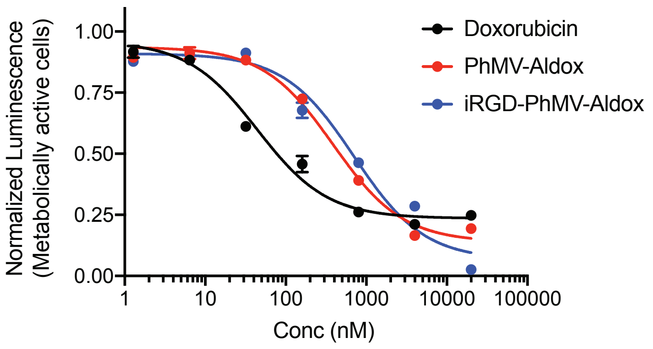

Supplemental Figure 10. Cytotoxicity of PhMV-Aldox and iRGD-PhMV-Aldox. Cytotoxicity of PhMV-Aldox and iRGD-PhMV-Aldox in A2780 cells as compared to molar equivalent dose of free doxorubicin. Error bars represent the S.E.M. of three replicates.

Supplemental Figure 11

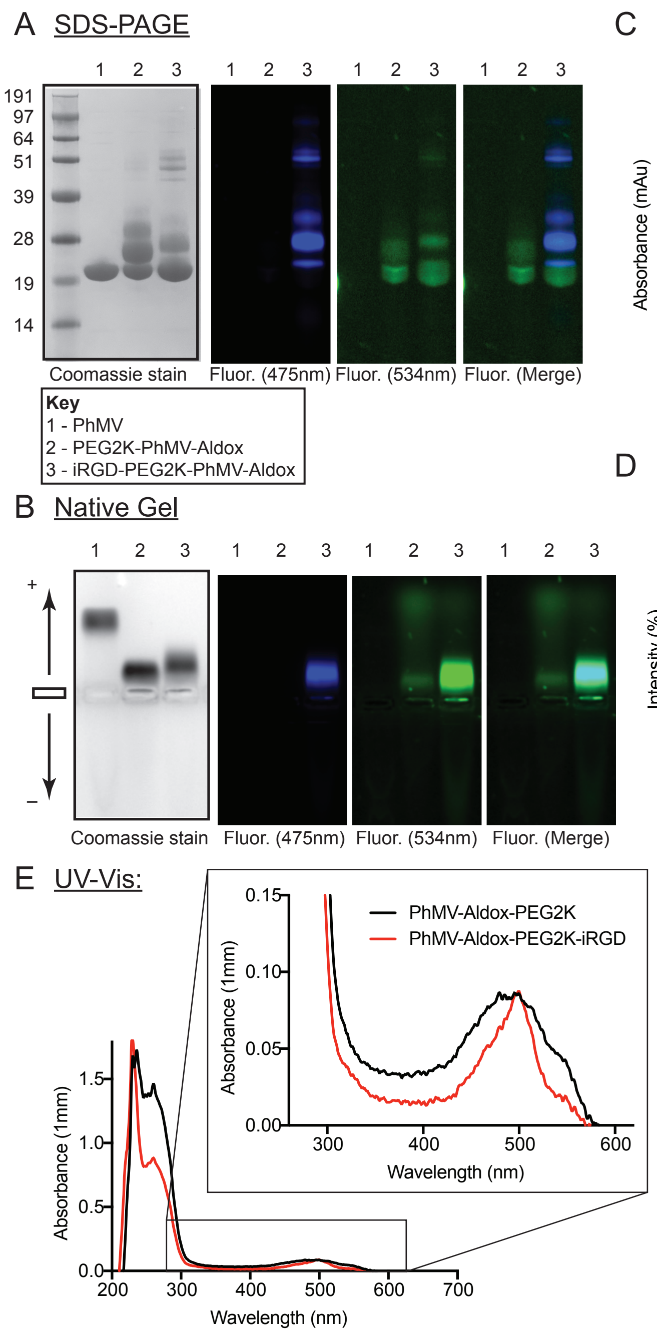

Supplemental Figure 11. Synthesis of iRGD-PEG2K-PhMV-Aldox. A-E. Characterization of PEG2K-PhMV-Aldox and iRGD-PEG2K-PhMV-Aldox by SDS-PAGE (A), native gel electrophoresis (B), size-exclusion chromatography (C), dynamic-light scattering (D), and UV-Vis spectroscopy (E). Error bars represent the S.E.M. of three replicates.

Supplemental Figure 12

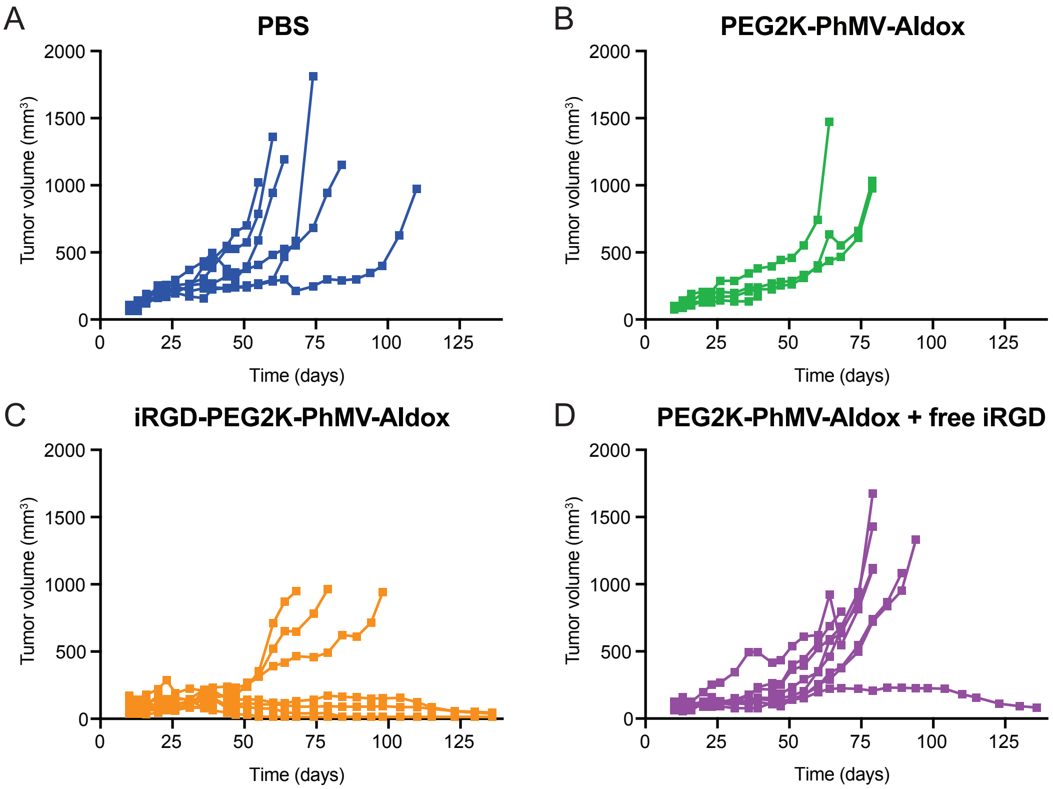

Supplemental Figure 12. Inhibition of growth in an MDA-MB-231 xenograft model. A-D. Individual tumor sizes in PBS (A), PEGK2K-PhMV-Aldox (B), PEG2K-PhMV-Aldox + free iRGD (C), and iRGD-PEG2K-PhMV-Aldox (D) treatment groups.
